# A Silicon-on-Insulator-Based Dual-Gain Charge-Sensitive Pixel Detector for Low-Noise X-ray Imaging for Future Astronomical Satellite Missions

**DOI:** 10.3390/s18061789

**Published:** 2018-06-01

**Authors:** Sumeet Shrestha, Shoji Kawahito, Hiroki Kamehama, Syunta Nakanishi, Keita Yasutomi, Keiichiro Kagawa, Nobukazu Teranishi, Ayaki Takeda, Takeshi Go Tsuru, Ikuo Kurachi, Yasuo Arai

**Affiliations:** 1 Research Institute of Electronics, Shizuoka University, Hamamatsu, Shizuoka 432-8011, Japan; sumeet@idl.rie.shizuoka.ac.jp (S.S.); snakani@idl.rie.shizuoka.ac.jp (S.N.); kyasu@idl.rie.shizuoka.ac.jp (K.Y.); kagawa@idl.rie.shizuoka.ac.jp (K.K.); teranishi@circus.ocn.ne.jp (N.T.); 2 Information and Communication Systems Engineering, National Institute of Technology, Okinawa College, Okinawa 905-2171, Japan; hkame@okinawa-ct.ac.jp; 3 Department of Applied Physics and Electronic Engineering, University of Miyazaki, Miyazaki 889-2192, Japan; takeda@astro.miyazaki-u.ac.jp; 4 Department of Physics, Kyoto University, Kyoto 606-8502, Japan; tsuru@cr.scphys.kyoto-u.ac.jp; 5 High Energy Accelerator Research Organization, Tsukuba, Ibaraki 305-0801, Japan; kurachii@post.kek.jp (I.K.); yasuo.arai@kek.jp (Y.A.)

**Keywords:** active pixel sensor, charge sensitive amplifier, dual gain, high energy astrophysics, SOI pixel, spectroscopic performance, X-ray detector

## Abstract

In this paper, we report on the development of a monolithic active pixel sensor for X-ray imaging using 0.2 µm fully depleted silicon-on-insulator (SOI)-based technology to support next generation astronomical satellite missions. Detail regarding low-noise dual-gain SOI based pixels with a charge sensitive amplifier and pinned depleted diode sensor structure is presented. The proposed multi-well sensor structure underneath the fully-depleted SOI allows the design of a detector with low node capacitance and high charge collection efficiency. Configurations for achieving very high charge-to-voltage conversion gain of 52 µV/e^−^ and 187 µV/e^−^ are demonstrated. Furthermore, in-pixel dual gain selection is used for low-noise and wide dynamic range X-ray energy detection. A technique to improve the noise performance by removing correlated system noise leads to an improvement in the spectroscopic performance of the measured X-ray energy. Taken together, the implemented chip has low dark current (44.8 pA/cm^2^ at −30 °C), improved noise performance (8.5 e^−^ rms for high gain and 11.7 e^−^ rms for low gain), and better energy resolution of 2.89% (171 eV FWHM) at 5.9 keV using ^55^Fe and 1.67% (234 eV FWHM) at 13.95 keV using ^241^Am.

## 1. Introduction

Recent decades have witnessed considerable progress in the use of X-ray imaging and spectroscopy to enhance our understanding of the complexities of the universe, such as new insights into black holes, supernova remnants, and the evolution of stars through cosmic time of galaxies [1]. X-ray detectors are considered to be fundamental and key components to any space missions. High energy astrophysics requires a detector with wide pass-band spectral response and high hit position pixel readout time.

Most astronomical satellites employ charge-coupled device (CCD) detectors for X-ray imaging (e.g., Swift’s X-ray telescope, Chandra’s advanced CCDs imaging spectrometer, Suzaku’s X-ray imaging spectrometer, XMM-Newton’s European photon imaging camera) [2,3]. A CCD X-ray detector offers good spatial resolution for large format devices with wide field of views and has very linear behavior over the range of incident energy [4]. The CCD detector technology for space has reached its maturity with a near fano-limited energy resolution, high quantum efficiency, and low readout noise. However, at high event count rates, CCDs with large array are limited in their ability to measure X-rays accurately. Due to the photon pile-up effect, multiple X-ray events can occur at the same pixel, thus limiting the energy resolution and other electro-optical performance. Detecting the target hit pixel with an X-ray event facilitates fast readout with no pile-up. An active pixel sensor (APS) which has an amplifier in each pixel and each column provides the opportunity for window readout and also requires much lower bandwidth per amplifier. The APS is also less sensitive to radiation damage as the generated charge travels less than 200 µm within the detector substrate, unlike CCDs where the charge travels multiple centimeters.

To exploit the APS performance reaching almost similar to, if not better than, CCD detectors and with the added performance benefit, rapid worldwide research development for replacing future X-ray imagers using the aspects of APS technology is currently ongoing. The most recent hybrid APS CMOS detector (H2RG) developed at Pennsylvania State University with Teledyne Imaging Sensors has demonstrated an energy resolution of 156 eV at 5.9 keV [5]. Another hybrid detector from MIT Lincoln Laboratory uses 3-D integration of silicon diode array with CMOS readout circuitry on a separate silicon-on-insulator (SOI) wafer [6]. Hybrid sensors allow for design flexibility to optimize the detector and readout circuitry separately. However, crosstalk between adjacent pixels due to inter-pixel capacitance caused by extra bonding (bond bonding or 3-D via) required between a detector and readout circuits has shown to adversely affect the overall detector performance and necessitate mathematical or circuit complication to minimize the effect. Additionally, MPI Semiconductor Laboratory has demonstrated the DEPFET monolithic pixel detector for high energy particle imaging [7]. Another monolithic detector optimized for X-ray detection is being developed at the Smithsonian Astrophysical Observatory with SRI/Sarnoff [8]. These monolithic X-ray detectors have shown low noise performance. However, these monolithic pixel detectors are optimized for different applications, resulting in a tradeoff between depletion depth (10 μm in Sarnoff) and pixel size (~70–100 μm in MPI DEPFET) for the optimal detector performance [4].

Authors are developing an X-ray detector called XRPIX using an SOI-based monolithic pixel detector (SOIPIX) technology led by high energy accelerator research organization, KEK in collaboration with Lapis semiconductor co. ltd [9,10,11]. The SOIPIX leverages the advantages of monolithic pixel detectors with high-resistivity silicon substrate used as a detector and SOI CMOS readout circuits implemented on thick insulating oxide layer. Both the SOI circuit and detector can be optimized independently for achieving the best performance for low-noise wide-dynamic-range X-ray detection. The fully depleted thick sensing region of the handle substrate is suitable for a wide range of high energy imaging [3,4] and on-chip integration of very fast low-noise readout circuits is used for high throughput.

Several productions of the XRPIX series and tests of their performance have been reported [11,12,13,14,15,16,17]. We have achieved improved spectroscopic performance using in-pixel charge sensitive amplifier (CSA). In Ref. [16], pixels with the CSA demonstrated an energy resolution of 5.4% FWHM at 5.9 keV with readout noise of 35 e^−^ rms. In Ref. [17], we have introduced an SOI pixel detector using pinned depleted diode (SOIPIX-PDD) structure. The X-ray spectrum and noise performance are greatly improved by using the PDD structure underneath the SOI circuits. In this paper, we present a SOIPIX-PDD-based pixel using dual-gain amplification using two feedback capacitors in a CSA for realizing very high (>150 μV/e^−^) and moderate (~50 μV/e^−^) conversion gains and resulting low-noise and wide-dynamic-range detection. A system-noise reduction technique is also introduced to further reducing the noise below our target requirement (<10 e^−^ rms) and very-low readout noise is achieved at high conversion gain. Evaluation results from ^55^Fe and ^241^Am X-ray sources using the SOIPIX-PDD based dual-gain CSA pixel with improved X-ray spectroscopic performance are discussed.

## 2. SOIPIX-PDD

**Figure 1:**
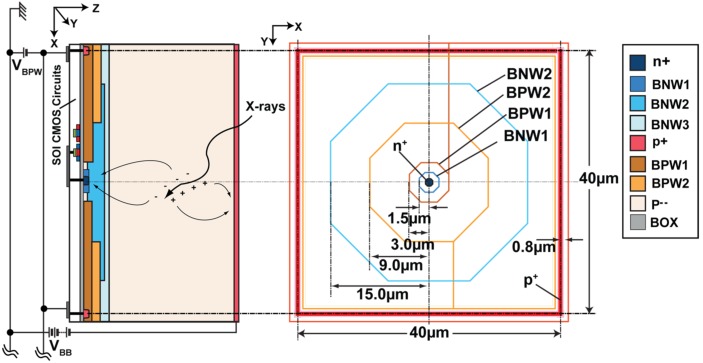
Proposed SOIPIX-PDD with multi-well structure under SOI and its layout. SOIPIX-PDD uses a 0.2 μm low-leakage fully-depleted SOI CMOS process. BNW1, BNW2, and BNW3 are buried n-wells with different doping concentrations. BPW1 is the buried p-well underneath the circuit pinned to a fixed negative voltage. BOX is the thick buried oxide insulting layer.

The SOI pixel technology allows us to incorporate a highly sensitive, high speed, low capacitance detector and CMOS circuits in a single chip. Figure 1 shows the SOIPIX-PDD along with layout pattern for multi-well structure. Fabrication of SOIPIX-PDD follows the conventional SOI process to form the top SOI CMOS circuit. Buried oxide (BOX) is then opened to implant the p^+^ and n^+^ dopant in the detector substrate. Also, the contact is formed through BOX [10]. Then, a special backend process is implemented to create the nested well-structure for SOIPIX-PDD. The designed prototype chip has pixel size of 40 µm × 40 µm. The proposed detector uses high-resistivity substrate (>25 kΩcm) with a thick depletion layer of 200 μm for achieving high quantum efficiency for the detection of high energy X-rays (e.g., >10 keV) [18,19]. Also, the SOIPIX-PDD structure is used for low-noise and high charge collection efficiency [17].

High negative voltage is applied at the backside of the detector to fully deplete the substrate. The fully-depleted region, except for the charge detector (n^+^), creates a very low node capacitance. A low capacitance at the charge-detector node is required to obtain low readout noise [17]. In a previous structure [16], applying a very high backside voltage adversely affected the CMOS circuit performance with a threshold voltage (V_th_) shift and crosstalk between the sensing node and SOI CMOS circuits, termed as the back-gate effect. The BPW1 is introduced under the thick oxide to isolate the charge detector and top SOI circuit [10]. Pinning the surface to a fixed potential eliminates the V_th_ shift [17]. Any changes in the detector substrate will not affect circuit performance. Also, it reduces dark current generation by filling the interface traps with relatively high-density holes of the BPW1 at the Si surface. The nested n-well (BNW1, BNW2, and BNW3) structure forms a lateral electric field.

**Figure 2:**
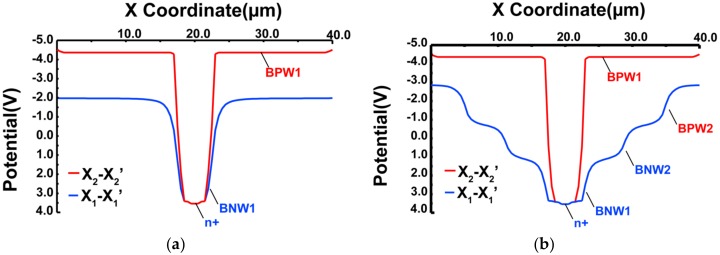
Horizontal potential profile simulations. (**a**) Simulated potential profiles of SOIPIX-PDD along X_2_-X_2′_ and X_1_-X_1′_ horizontal cross sections in absence of BNW2 and BPW2. (**b**) Simulated potential profiles of SOIPIX-PDD along X_2_-X_2′_ and X_1_-X_1′_ horizontal cross sections with multiple wells.

The potential profile simulation of the 40 µm × 40 µm pixel detector along horizontal cross-sections X_1_-X_1′_ and X_2_-X_2′_ are shown in Figure 2 to compare the advantage of a nested multiple-well structure of SOIPIX-PDD. X_2_-X_2′_ is a horizontal cross-section near the detector just under BOX layer and X_1_-X_1′_ is at a depth of Z = 0–10 μm. Figure 2a shows the lateral electric field with SOIPIX-PDD without BNW2 and BPW2 wells. The shape of the potential profile at X_1_-X_1′_ indicates that the charge collection area is small which reduces the charge collection efficiency. By using BNW2 and BPW2 wells, a lateral electric field is created to increase the speed and area for charge collection. The step-wise lateral electric fields created by each well are indicated in Figure 2b. The lateral electric field is used for providing a fast and easy path for the charge to be collected at the detector node. It helps in increasing the charge collection efficiency near the surface.

**Figure 3:**
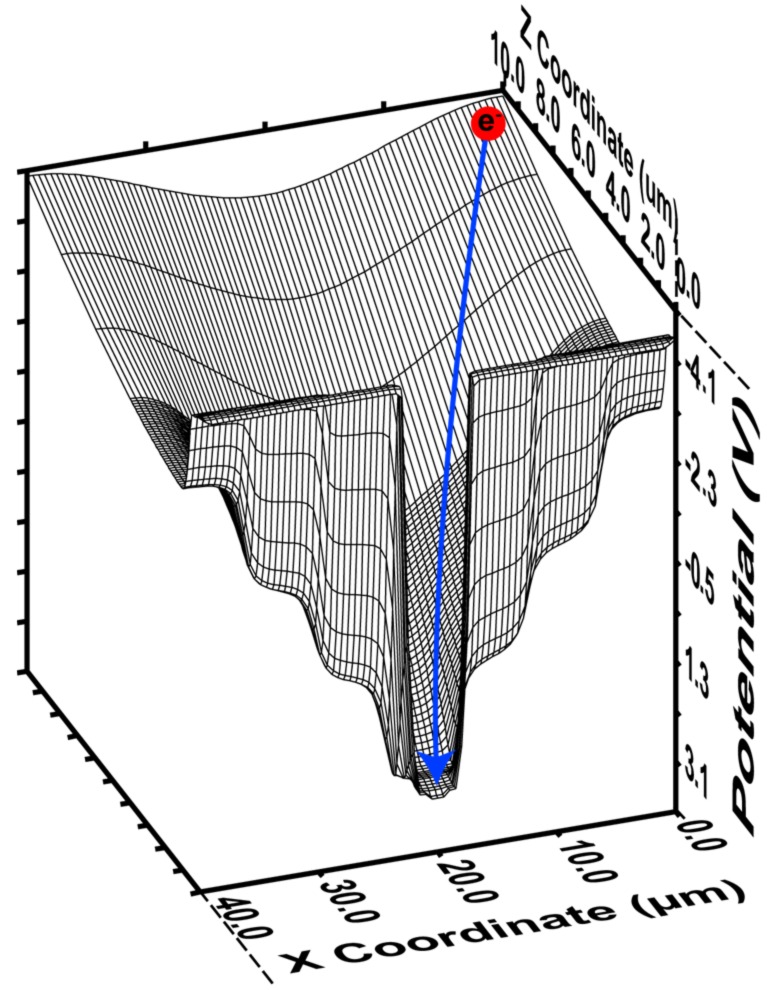
3D potential profile simulation of the SOIPIX-PDD with 40 μm pixel pitch. The blue line shows that an expected generated charge will be collected at charge detector node.

The simulated potential profile plot along *X* and *Z*-coordinates of SOIPIX-PDD near the surface (Z = 0 to 10 μm) is shown in Figure 3. Node voltage of 2.5 V at n^+^ and BPW1 pinned potential of −4 V is applied. The step-wise graded electric filed created near the surface of the detector isolates each pixel charge collection area. Charges generated in the detector substrate are accelerated faster towards sense node assisted by a vertical and a lateral electric field. With this profile, all the charges generated at the arbitrary position in the pixel will be successfully collected into the charge detector. In the event of X-ray absorption between two pixels, each pixel may share the part of the total generated charge. Collected charges from each pixel should be considered in the case multi-pixel X-ray hit event. The statistical noise from two or more pixels may degrade the X-ray spectrum. In this paper, an X-ray passing via two or more neighboring pixels is excluded and only single pixels with X-ray hits are considered in X-ray spectra analysis for an accurate measurement of detector performance.

**Figure 4:**
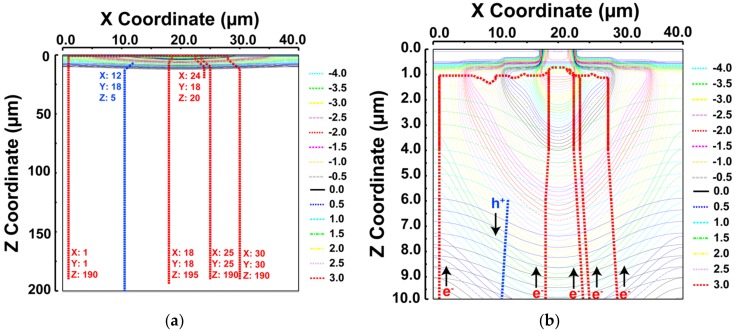
Equipotential plot. (**a**) Electron and hole path for Z = 0–200 μm. (**b**) Electron and hole path for Z = 0–10 μm. Electrons are randomly placed at (1, 1, 190), (18, 18, 195), (24, 18, 20), (25, 25, 190), (30, 30, 190) and a hole is placed at (12, 18, 5).

In order to confirm the potential path of generated charge, electrons are randomly placed in 3D space at (X, Y, Z) = (1, 1, 190), (18, 18, 195), (24, 18, 20), (25, 25, 190), (30, 30, 190) and a hole is placed at (12, 18, 5). As shown in the equipotential plot and carrier movement of Figure 4, electrons generated within the detector substrate are pushed towards the detector node (n^+^) and holes drift to the backside of the detector. Figure 4a shows the charge path within a substrate depth of 0 to 200 μm and Figure 4b is a zoomed view within depth of 0 to 10 μm.

## 3. Dual-Gain Pixel with a Charge Sensitive Amplifier

**Figure 5:**
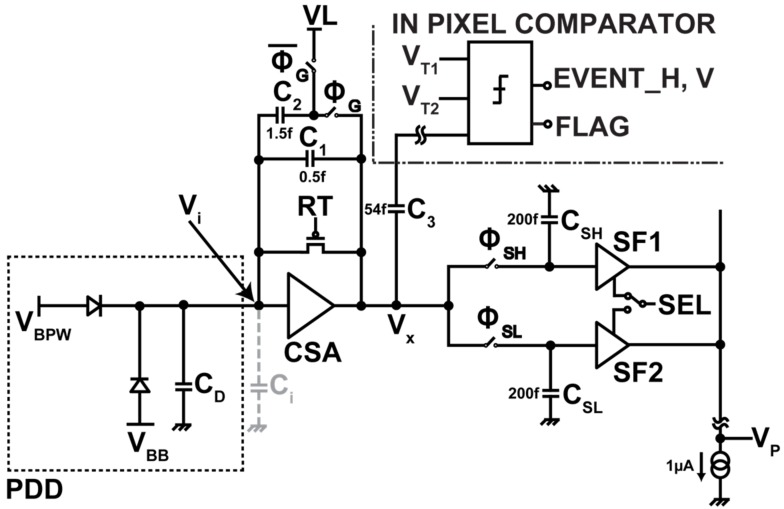
Dual-gain CSA pixel circuit. CSA is an in-pixel charge-sensitive amplifier. RT is the reset transistor. C_1_ = 0.5 fF and C_2_ = 1.5 fF are two feedback capacitors. C_SH_ and C_SL_ are sampling capacitors for high and low gain, respectively. SF1 and SF2 are source followers. SEL is a selector switch to choose one of the outputs for readout.

**Figure 6:**
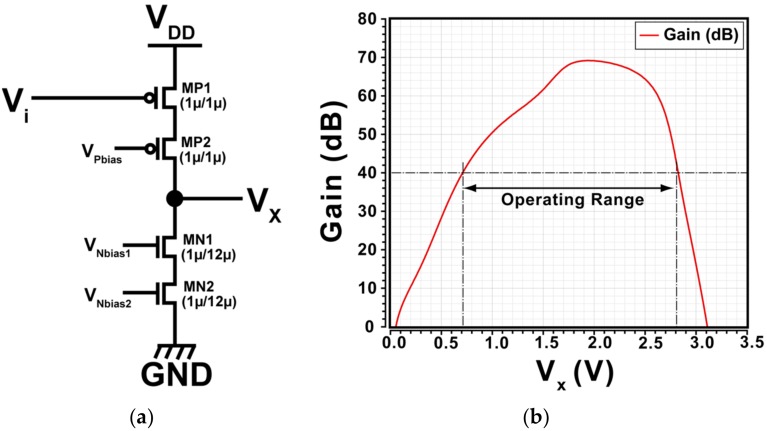
(**a**) Internal amplifier (cascode common-source amplifier). (**b**) Gain characteristics with output voltage. Operating range (gain > 40 dB) is 0.7 V to 2.8 V.

The design of pixel circuits is very important for realizing a low-noise and wide dynamic range detector. We have developed a pixel circuit with the dual-gain charge sensitive amplifier (CSA). Figure 5 shows a unit pixel with the CSA and selective feedback capacitors (C_FB_: C_1_ and C_2_) for the dual in-pixel gain. Figure 6a,b shows the internal amplifier used in the CSA and its gain characteristics to output voltage (V_x_). With this simple circuit topology, an open-loop DC gain of over 40 dB for the operating range of 0.7 V to 2.8 V (voltage swing of 2.1 V) is obtained.

**Figure 7:**
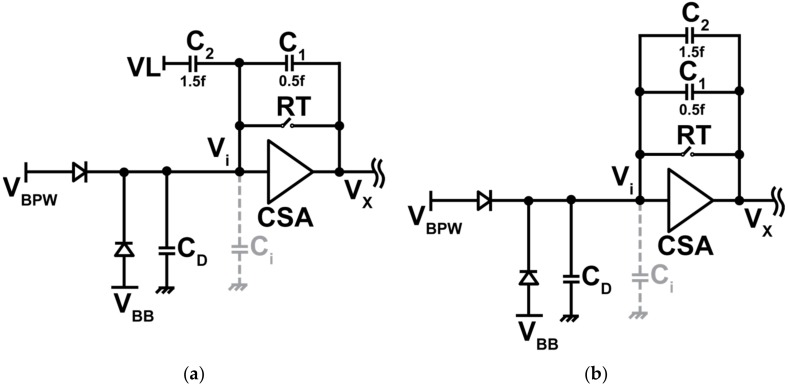
Low- and high-gain configurations. (**a**) Φ_G_ = 0, high-gain setting. Total feedback capacitor is 0.5 fF (C_FB_ = C_1_) (**b**) Φ_G_ = 1, low-gain setting. Total feedback capacitor is 2 fF (C_FB_ = C_1_ + C_2_).

Two different configurations are possible for the selection of feedback capacitors C_1_ (0.5 fF) and C_2_ (1.5 fF) using the switch Φ_G_ to realize two selectable conversion gains (G_C_s). The conversion gains for two cases of Figure 7a,b are given by:

Case 1: Φ_G_ = 1

(1) $$\;G_{C1}=\;\frac{G_{AMP}.q}{C_{t}+\;(C_{1}+C_{2}).G_{AMP}}\;\approx \;\frac{q}{C_{1}+C_{2}}\;\left(For,\;G_{AMP}\gg 1\right),$$

Case 2: Φ_G_ = 0

(2) $$\;G_{C2}=\;\frac{G_{AMP}.q}{C_{t}+\;(C_{1}).G_{AMP}+C_{2}}\;\approx \;\frac{q}{C_{1}}\;\left(For,\;G_{AMP}\gg 1\right),$$

and where, *G_AMP_* is the open loop DC gain of the CSA, *q* is the elementary charge (≈1.602 × 10^−19^) in coulomb, *C_t_* is the total parasitic capacitance at the input of the charge amplifier which includes the PDD detector capacitance (C_D_) and the input capacitance of the internal amplifier (C_i_). As shown in Equations (1) and (2), the conversion gain of the pixel can be solely determined by C_FB_ for the given configuration if *G_AMP_* is large enough. By using C_1_ = 0.5 fF and C_2_ = 1.5 fF, the expected conversion gains are *G_C_*_1_ of 80 μV/e^−^ and *G_C_*_2_ of 320 μV/e^−^. In the actual implementation, due to the parasitic capacitances and the attenuation in the overall readout signal pass consisting of the pixel, column circuits and output buffers, the actual conversion gains are smaller than these ideal values, but sufficiently large conversion gains of *G_C_*_1_ > 50 μV/e^−^ and *G_C_*_2_ > 150 μV/e^−^ are expected.

The operating point of the input of the CSA, V_i_* is set to a relatively high voltage for biasing high reversed voltage to the pn-junction diode between BPW1 and the n^+^ node.

By using a PMOS input transistor as shown in Figure 6a, the input biasing voltage (V_i_*), which is determined by the voltage when the CSA is reset by turning the RT on, is given by

(3) $$V_{i}^{*}=V_{DD}-\left|V_{od}\right|-\left|V_{TH,P}\right|$$

and where *V_DD_* is the supply voltage and *V_od_* and *V_TH,P_* are the overdrive voltage and threshold voltage of the PMOS input transistor, respectively. If the supply voltage (*V_DD_*) is 3.3 V, the overdrive voltage (*V_od_*) is 0.15 V and the threshold voltage of PMOS (*V_TH,P_*) is −0.5 V, the input biasing voltage V_i_* will be 2.65 V.

The designed amplifier has its linear operating region from 0.7 V to 2.8 V with high DC gain (>100) as shown in Figure 6b, but the actual available dynamic range depends on the initial output voltage before charge detection. The CSA output voltage increases upon charge detection and stores the charge in C_FB_ because the signal detected at the detector node is electron charge carrier.

**Figure 8:**
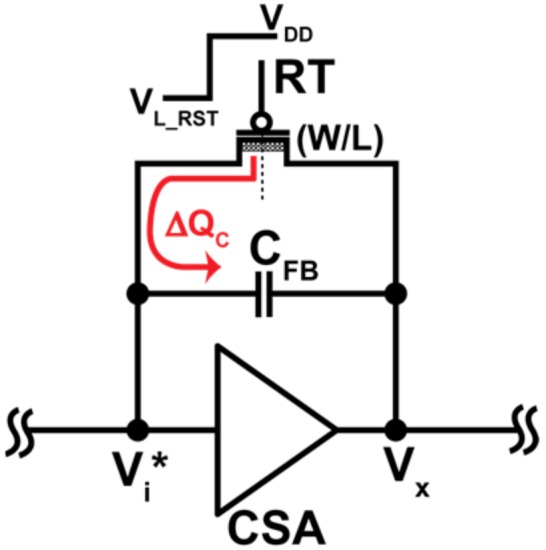
Charge injection from a reset transistor. The reset voltage on the reset transistor changes from *V_L_RST_* to *V_DD_*. ∆Q is the amount of charge injection after the reset switch is turned OFF.

In order to insure the wide operating range of the CSA, the initial output voltage should be set at a level as low as possible (but higher than the lowest linear level of 0.7 V). To do this, a technique using the charge injection from the PMOS reset transistor to bring the voltage at V_X_ node to the desired lower level is proposed. A charge injection model during reset is shown in Figure 8. As charge injection is the dominant factor determining the output voltage of the CSA, the charge sharing due to clock feedthrough in the reset switch is ignored. Voltage at the gate of reset transistor is changed from *V_L_RST_* to *V_DD_* after reset. Half of the generated charge (∆*Q_C_*) from the reset transistor is injected back to the input node. Charge injection from the reset transistor can be controlled by using a proper size (*L*, *W*) and applied gate voltage to the reset transistor and is given by

(4) $$\Delta Q_{C}=\;\frac{1}{2}C_{OX}WL\left(\left|V_{GS}\right|-\left|V_{TH,P}\right|\right)\;=\frac{1}{2}C_{OX}WL\left(V_{i}^{*}-V_{L\_RST}-\left|V_{TH,P}\right|\right)\;$$

where *C_OX_* is the unit gate capacitance per area of the reset transistor. With this level shifting technique, the initial output voltage after reset, V_x_* is given by

(5) $$\;V_{x}^{*}=V_{i}^{*}-\Delta Q_{C}/C_{FB}$$

With the parameters of *C_OX_WL* = 3.6 fF, *C_FB_* = 2 fF, *V_L_RST_* = 0 V, *V_TH,P_* = −0.5 V, and V_i_* = 2.65 V, for instance, V_x_* can be set at 0.715 V.

**Figure 9:**
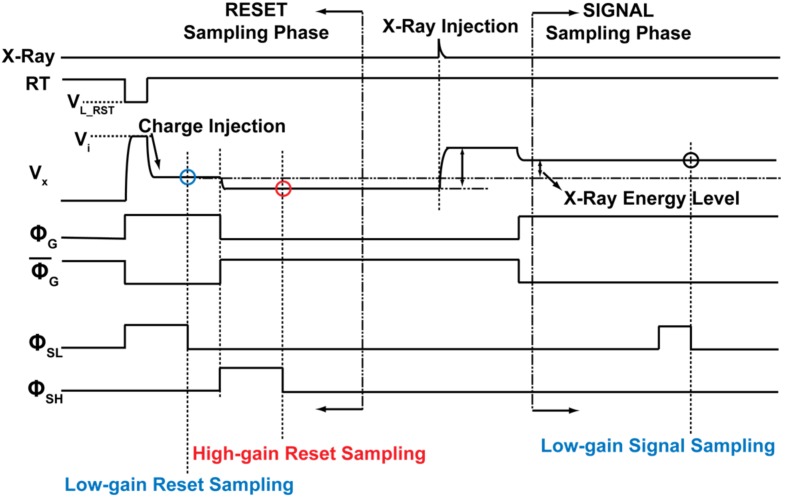
Pixel timing diagram. RT is the reset pulse. V_X_ is the voltage at CSA output node. $\Phi_{G}$ and $\bar{\Phi_{G}}$ are gain selection switches. $\Phi_{\mathrm{SL}}$ and $\Phi_{\mathrm{SH}}$ are sampling switches for low and high gain, respectively.

The pixel level timing diagram shown in Figure 9 is used in conjunction with readout operation. An individual pixel has a signal processing unit for dual-gain selection and an event detection unit with an in-pixel comparator for event triggered operation. Both low and high gain reset levels are sampled during the reset phase. The pixel can be operated in frame readout or event readout mode. In event readout mode, arrival of X-ray energy greater than the minimum detectable energy is detected by scanning a signal from the event triggered (in-pixel comparator circuit) and scanning circuit (event scanner). Classification of the X-ray into low and high energy levels is done at the trigger circuit and is used for the selection of in-pixel gain. In frame readout mode, a predetermined gain is applied to the input X-ray energy. Signals are sampled with 1 ms accumulation time for both low and high gain. In this paper, we present the results with frame readout mode for characterizing the detector’s basic performance.

**Figure 10:**
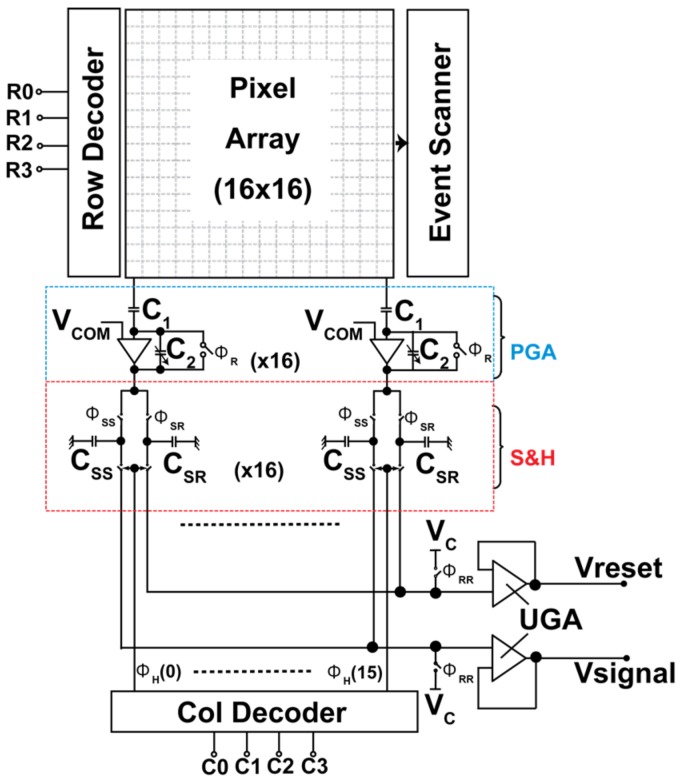
Sensor architecture. R0–R3 are row selectors, C0–C3 are column selectors, V_COM_ and V_C_ are common mode voltages of the column amplifier and output buffer, respectively.

Figure 10 shows the designed sensor’s architecture. Four-bit row and column decoders are used to access an individual pixel. The readout circuit consists of the column amplifier with programmable gain (PGA), the sample and hold circuit (S&H) for correlated double sampling (CDS), and the output unity gain buffer amplifier (UGA).

## 4. Low-Noise Design and System-Noise Reduction

Low-noise performance is important for any future X-ray satellite missions. Continued innovation in the detector and circuit design is crucial to improve the noise performance for better X-ray spectroscopy. Introduction of the CSA pixel circuit with dual-gain pixels is used to achieve the target requirement noise level (<10 e^−^ rms) and a higher dynamic range.

**Figure 11:**
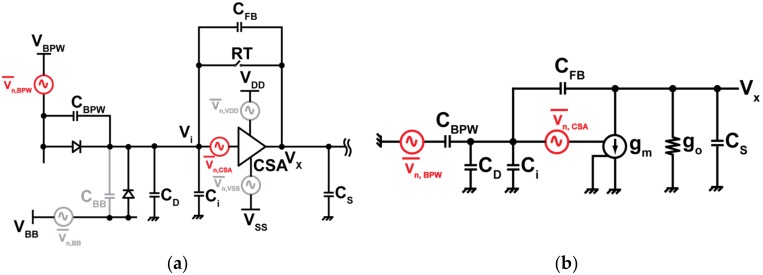
Equivalent noise model of CSA SOIPIX-PDD circuit. (**a**) Circuit with noise sources from BPW1, internal amplifier, VBB, and power line. (**b**) Simplified small signal noise model of pixel circuit. C_BPW_ is the capacitance due to BPW1, C_D_ is the detector capacitance, C_i_ is the input capacitance of the internal amplifier, C_S_ is the sampling capacitor for low or high output from the amplifier.

The main sources of the noise in the pixel circuit are from the internal in-pixel charge amplifier and the noise from the system coming from the power lines. Figure 11a shows the noise source in the CSA pixel circuit. The noise of the designed pixel, *N_n_*, expressed as the equivalent number of electrons is given by

(6) $$N_{n}=\;\frac{\sqrt{2}}{G_{C}}\;\sqrt{{\left(1+\frac{C_{D}+C_{FB}+C_{i}}{C_{FB}}\right)}^{2}\bar{V_{n,CSA}^{2}}+{\left(\frac{C_{BPW}}{C_{FB}}\right)}^{2}\bar{V_{n,BPW}^{2}}+{\left(\frac{C_{BB}}{C_{FB}}\right)}^{2}\bar{V_{n,BB}^{2}}\;+\frac{1}{PSRR^{2}}\left(\bar{V_{n,VDD}^{2}}+\bar{V_{n,VSS}^{2}}\right)}$$

where, $\bar{V_{n,CSA}^{2}}$ is mean square noise from the internal amplifier, $\bar{V_{n,BPW}^{2}}$ is the mean square noise coupled by large buried p-well in the surface, $\bar{V_{n,BB}^{2}}\;$ is the mean square noise from the back-gate voltage, $\bar{V_{n,VDD}^{2}}$ and $\bar{V_{n,VSS}^{2}}\;$ are the mean square system-noise from the supply voltages of the power lines, and *PSRR* is the power-supply rejection ratio of the CSA. Assuming the CSA is designed to have high PSRR, and the capacitance, C_BB_, is very small because of very high reversed bias, the noises coming from these sources ($\bar{V_{n,BB}^{2}},\;\bar{V_{n,VDD}^{2}},\;\bar{V_{n,VSS}^{2}}$) are ignored in our analysis. $\bar{V_{n,BPW}^{2}}$ is due to capacitive coupling at the detector node by a large BPW near the detector sense node. This system-noise becomes critical when using in-pixel CSA, as the noise coupled at the sense node will be increased by the equivalent gain factor (*C_BPW_/C_FB_*) of the charge amplifier. Figure 11b shows the small signal noise model for the CSA pixel circuit, showing the noise generated by the internal amplifier and BPW1 and thus Equation (6) can be simplified as

(7) $$\;N_{n}\approx \;\frac{\sqrt{2}}{G_{C}}\;\sqrt{{\left(1+\frac{C_{D}+C_{FB}+C_{i}}{C_{FB}}\right)}^{2}\bar{V_{n,CSA}^{2}}+{\left(\frac{C_{BPW}}{C_{FB}}\right)}^{2}\bar{V_{n,BPW}^{2}}\;}\;$$

If the system-noise from BPW1 can be sufficiently reduced, the noise only from the amplifier dominated by thermal and flicker noise of the transistors in the amplifier [17] can be approximately expressed as

(8) $$N_{n,CSA}=\;\frac{\sqrt{2}}{G_{C}}\;\sqrt{\left(\frac{C_{FB}+C_{D}+C_{i}}{C_{FB}}\right)\frac{\xi_{A}k_{B}T}{C_{S}}+{\left(\frac{C_{FB}+C_{D}+C_{i}}{C_{FB}}\right)}^{2}N_{f}\left[\varepsilon +ln\left\{\left(\frac{C_{FB}+C_{D}+C_{i}}{C_{FB}}\right)\frac{C_{S}T_{CDS}}{g_{m}}\right\}\right]\;}$$

where, $\xi_{A}$ is the excess thermal noise factor of the amplifier, $k_{B}$ is the Boltzmann constant, *T* is the temperature in Kelvin, $N_{f}$ is the flicker noise coefficient and $g_{m}$ is the transconductance of the input transistor of the amplifier, ε is the Euler’s constant, and $T_{CDS}$ is the time difference between two samples in the correlated double sampling operation for the reset noise cancellation. The mean square noise from the amplifier ($\bar{V_{n,CSA}^{2}}$) is calculated as 5.7 e^−^ rms and 3.5 e^−^ rms for C_FB_ 2 fF and 0.5 fF, respectively [20]. The influence of system-noise increases the overall noise of the sensor. We can decrease the effect of the system-noise from each pixel if they are correlated. Since reset and signal sampling are done using a global signal and stored at in-pixel local sampling capacitors; correlated system level noise can be minimized by canceling the common noise level from individual pixels. Each pixel noise can be divided into readout noise (varies from pixel to pixel) and correlated system-noise (common to all the pixels).

**Figure 12:**
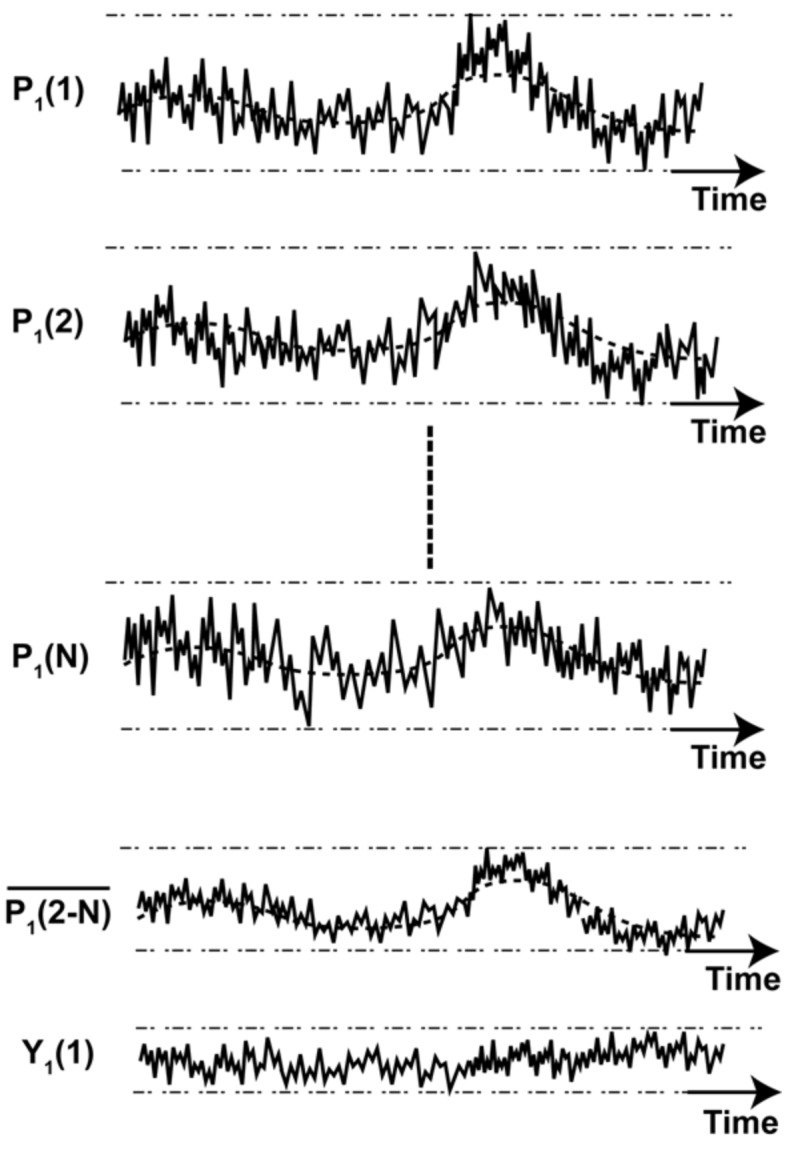
System-noise reduction. P_1_(1), P_1_(2), and P_1_(N) are hypothetical pixels with dominant system-noise in pixel 1, 2, and N, respectively, for frame 1. $\bar{P_{1}\left(2-N\right)}$ is the average of pixels from 2 to N. Y_1_(1) is the final pixel with reduced noise.

A hypothetical random noise from all pixels (1 to N) is shown in Figure 12 for the illustration of system-noise reduction. P_N_ (N = 1 to 96 (the total number of pixels)) is a pixel value after CDS and dark current offset correction. System-noise with relatively lower frequency components is assumed to be superimposed in all the pixels. Since the system-noise must be common for all the pixels, the noise component can be estimated by averaging over the pixels which does not receive signals or calculating the mean value of such pixels. The final pixel value (*Y_K_*(*i*)) for each frame (*K*) is calculated by subtracting the mean value of remaining pixels from the pixel of interest, i.e., it is given by Equation (9). The new system-noise reduced pixel value (*Y_K_*(*i*)) will have a smaller noise level compared to P_K_, as shown in Figure 12. The final rms noise with minimized system-noise containing mainly noise from the amplifier and pinned BPW is estimated using the mean and standard deviation given by Equations (10) and (11), respectively.

(9) $$Y_{K}\left(i\right)=P_{K}\left(i\right)-\frac{1}{N-1}\overset{N}{\underset{j=1}{\sum }}P_{K}\left(j\right)\;$$

(10) $$\bar{Y_{K_{N}}}=\;\frac{1}{M}\overset{N}{\underset{i=1}{\sum }}Y_{K_{N}}\left(i\right)\;$$

(11) $$\sigma_{N_{x}}=\sqrt{\frac{1}{M-1}\overset{M}{\underset{i=1}{\sum }}{\left(Y_{K_{N}}\left(i\right)-\bar{Y_{K_{N}}}\right)}^{2}}$$

Here, *N* is the number of pixels and *M* is the number of frames.

## 5. Results and Discussion

**Figure 13:**
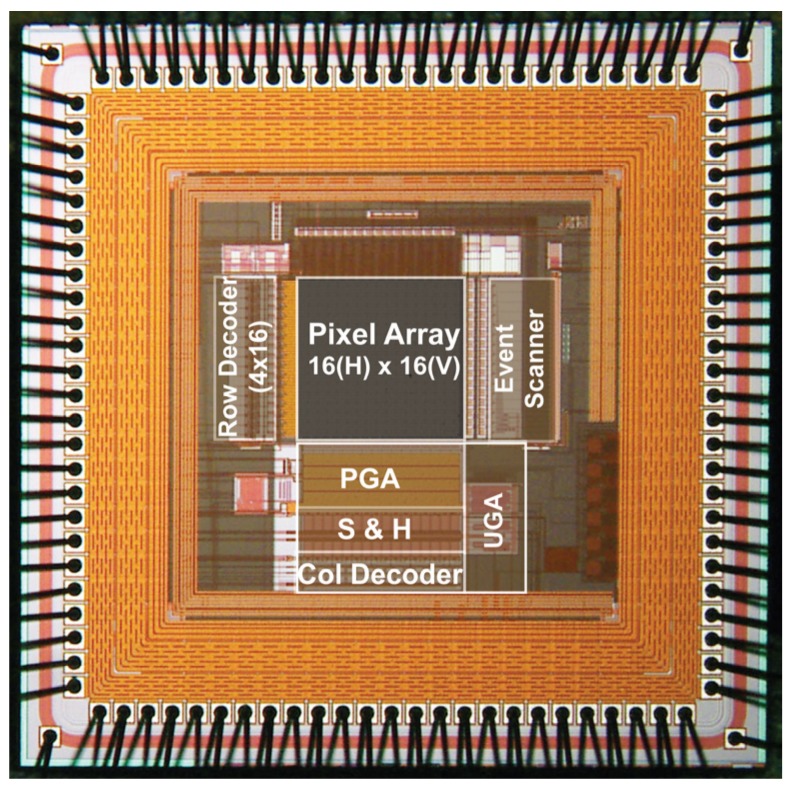
Chip micrograph of the design prototype SOIPIX-PDD detector. PGA—programmable gain column amplifier. S&H—sample and hold circuits. UGA—unity gain output amplifier.

The chip micrograph image of an experimental 16 × 16 SOIPIX-PDD detector is presented in Figure 13. The designed prototype chip has an effective number of 16 × 6 (out of 16 × 16) CSA-type pixel array with 40 μm pixel pitch. For measurement, the chip is mounted on an evaluation board with off-chip 14 bit ADC (AD9240) and FPGA (ALTERA Cyclone III).

### 5.1. Linearity and Conversion Gain

**Figure 14:**
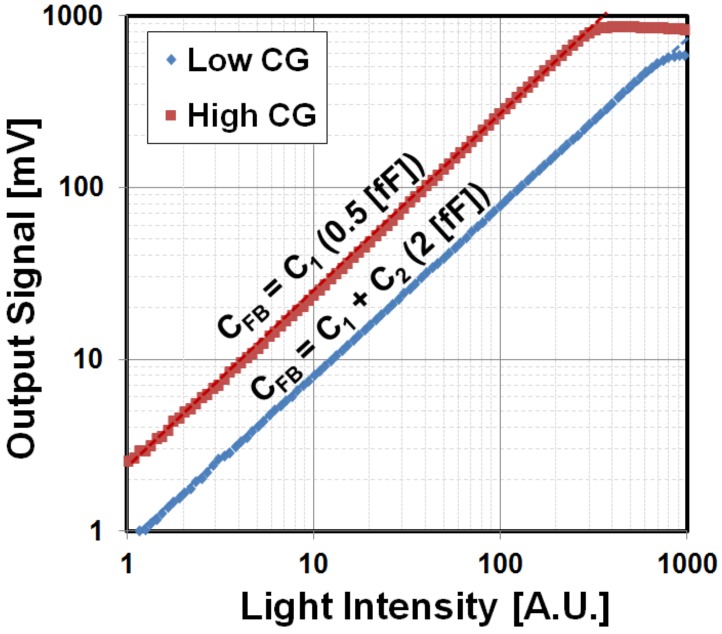
Linearity plot for low- and high-gain settings. The light intensity with different illuminance value (LUX) is changed to characterize the pixel output response for two C_FB_ (0.5 fF and 2 fF) configurations.

Gain can be selected using in-pixel C_FB_ configuration (Figure 7). The output responses for two different gains with C_FB_ 2 fF (low gain) and 0.5 fF (high gain) are shown in Figure 14. Output of the pixel for both low and high gains are linear with the input light intensity up to 0.6 V and 0.8 V, respectively.

**Figure 15:**
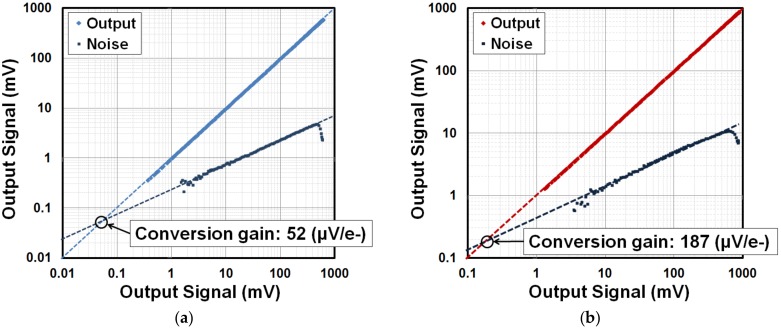
Photon transfer curve. (**a**) Low-gain setting, low conversion gain is 52 µV/e^−^ and (**b**) high-gain setting, high conversion gain is 187 µV/e.

The conversion gain (G_C_) for low- and high-gain settings is measured with the photon transfer curve plot with signal and noise (shot noise) obtained at different light intensity. Figure 15a,b shows the G_C_s of 52 µV/e^−^ and 187 µV/e^−^ measured for C_FB_ of 2 fF and 0.5 fF, respectively. This allows the designed sensor to detect X-ray energy up to 42 keV (0.6 V at 52 μV/e^−^) at low-gain setting and 15.6 keV (0.8 V at 187 μV/e^−^) at high-gain setting. The gain with 0.5 fF is 3.6 times greater than that with 2 fF. This ratio is smaller than our expected design value which may be due to an increase in the parasitic capacitance. The signal-to-noise ratio (SNR) of the circuit can be improved by using the high-gain setting for the incoming signal.

### 5.2. Dark Current Measurement

**Figure 16:**
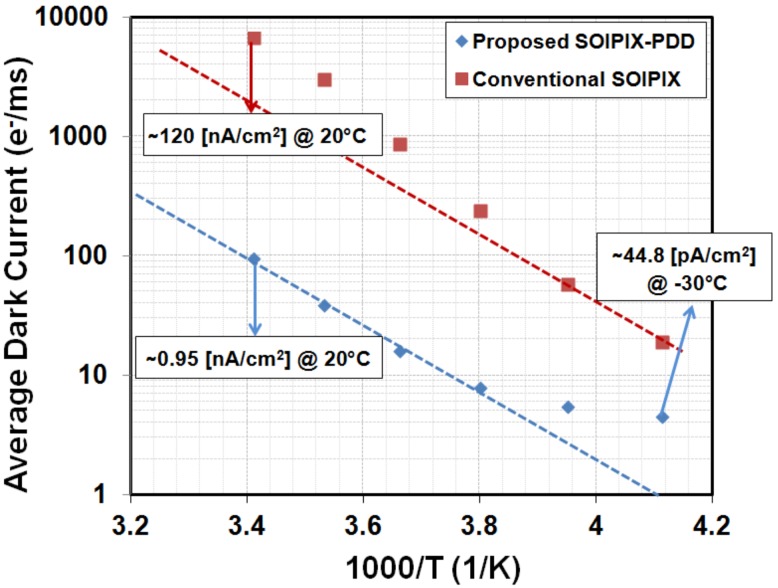
Temperature dependence of detector dark current. The average dark current from the proposed SOIPIX-PDD is compared with a conventional SOIPIX.

The temperature dependence of dark currents is shown as an Arrhenius plot in Figure 16. The slope of the extrapolated dashed line for the average dark current represents the activation energy of half the band-gap energy of silicon (0.56 eV). Dark current of conventional SOIPIX [11] is also plotted alongside for comparison. The average dark current of the SOIPIX-PDD detector is 100 times lower than the conventional SOIPIX at 20 °C because of the pinned depleted diode structure. A reduced dark current density of 44.8 pA/cm^2^ is observed at −30 °C.

### 5.3. X-ray Spectroscopic Performance

^55^Fe and ^241^Am radioisotope materials are used in our experiment for the characterization of the chip. Our experiment is conducted at a controlled temperature of −30 °C with a back-gate voltage of −60 V. An off-chip 14 bit A/D converter with analog output range of 2 V peak-peak is used. One analog to digital conversion unit (ADU) corresponds to 122 μV. X-ray events are measured using frame readout mode. X-rays that pass through two or more pixels are excluded and only a primary (central) pixel within 3 × 3 arrays with single X-ray events having a local maximum above the split threshold energy is retained for the analysis. The equivalent energy of the incident X-ray photon is estimated using the conversion gain (*G_C_*) and the ionization energy of Si (*ω* = 3.65 eV/e^−^) and is given by Equation (12). Energy resolution is estimated by fitting the peak of the obtained spectra with a Gaussian distribution. The standard deviation (*σ*) obtained from a Gaussian fit at the interest peak energy (E) is used to evaluate the full width half maximum (*FWHM*) and the energy resolution, and is given by Equations (13) and (14), respectively.

(12) $$Energy\;\left(eV\right)=\;\frac{Output\;\left(ADU\right)\;\times 122\;\mu V}{G_{C}}\omega \;$$

(13) $$FWHM\;\left(\Delta E\right)=\;2\sqrt{2ln2}\sigma \;$$

(14) $$Energy\;Resolution=\frac{\Delta E}{E}\times 100\%\;$$

**Figure 17:**
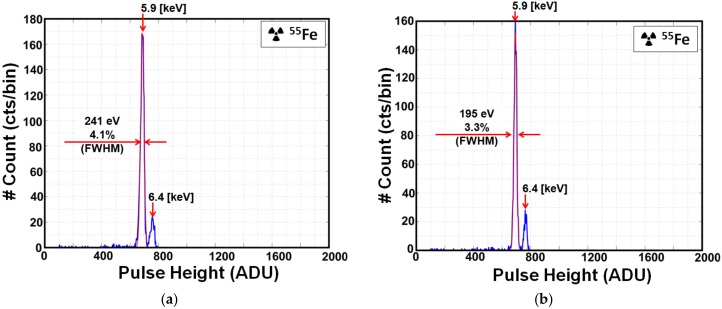
^55^Fe spectrum with a single X-ray event at 16 × 6 pixels using low-gain. (**a**) Spectrum of ^55^Fe before system-noise reduction and (**b**) spectrum of ^55^Fe after system-noise reduction. One bin = 3 ADU. Spectrum is fitted with a Gaussian distribution at the Mn-K_α_ (5.9 keV) line.

X-ray spectra are obtained from ^55^Fe using low- and high-gain settings. The ^55^Fe source has two characteristic peaks at 5.9 keV and 6.4 keV produced by Mn-K_α_ and Mn-K_β_ lines. For the spectrum obtained with a low-gain setting in Figure 17a, the Mn-K_α_ peak is obtained at 687 ADU, corresponding to 5.9 keV with G_C_ nearly equal to 51.8 μV/e^−^. Energy resolution is improved by the reduction of the overall noise using the system-noise reduction technique. As shown in Figure 17a,b, the energy resolution is improved to 3.3% (195 eV FWHM) at the Mn-K_α_ line after system-noise reduction when compared to 4.1% (241 eV FWHM) before system-noise reduction.

**Figure 18:**
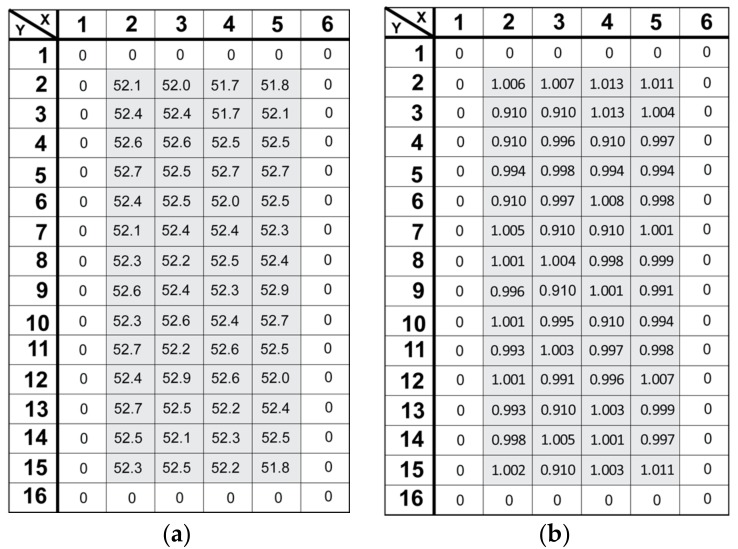
(**a**) Conversion gain for the low-gain setting estimated using ^55^Fe in the 16 × 6 pixel array. (**b**) Gain correction factor for 16 × 6 pixels. The average conversion gain is 52.4 μV/e^−^.

**Figure 19:**
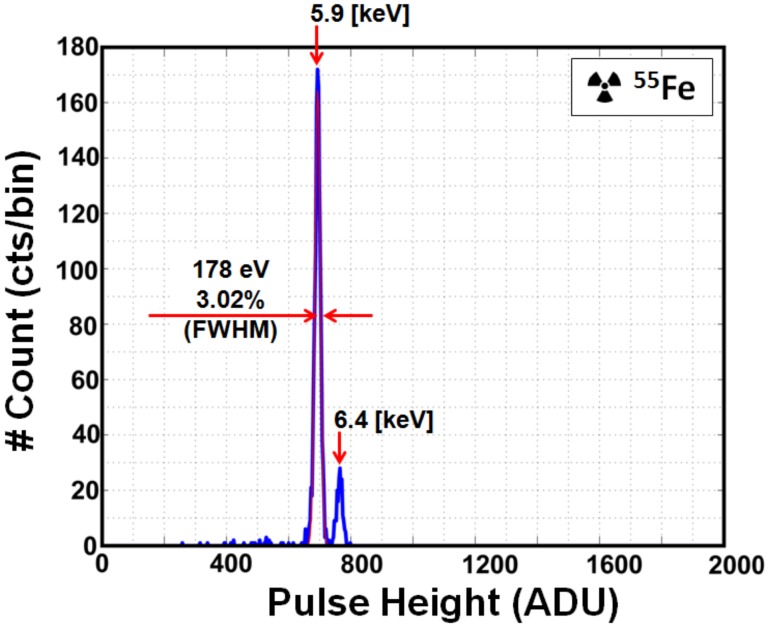
^55^Fe spectrum after noise reduction and gain correction at low-gain setting. One bin = 3 ADU. Spectrum is fitted with a Gaussian distribution at the Mn-K_α_ (5.9 keV) line. Energy resolution is 3.02% (178 eV FWHM) at the Mn-K_α_ line.

The result of Figure 17 contains the influence of pixel-to-pixel gain deviation. Purer spectra can be obtained if the pixel-to-pixel gain deviation is corrected. Figure 18a shows the conversion gains (μV/e^−^) for each pixel (except in the boundary elements). The spectrum of each pixel is evaluated using the X-ray source (^55^Fe) and individual pixel spectra were Gaussian fitted at the Mn-Kα line (5.9 keV) of ^55^Fe to evaluate the equivalent conversion gain. As shown in Figure 18a, each pixel within the 16 × 6 pixel array has deviation (standard deviation of 0.267 μV/e^−^) from the mean G_C_ value ($\bar{G_{C}}$ = 52.4 μV/e^−^). Figure 18b is the gain factor correction value for each pixel approximated using the ratio of mean value ($\bar{G_{C}}$ = 52.4 μV/e^−^) of gain to pixel gain. ^55^Fe spectrum after gain correction is plotted in Figure 19. The energy resolution is improved to 3.02% (178 eV FWHM) at 5.9 keV with G_C_ of 52.1 μV/e^−^ calculated at 691 ADU.

**Figure 20:**
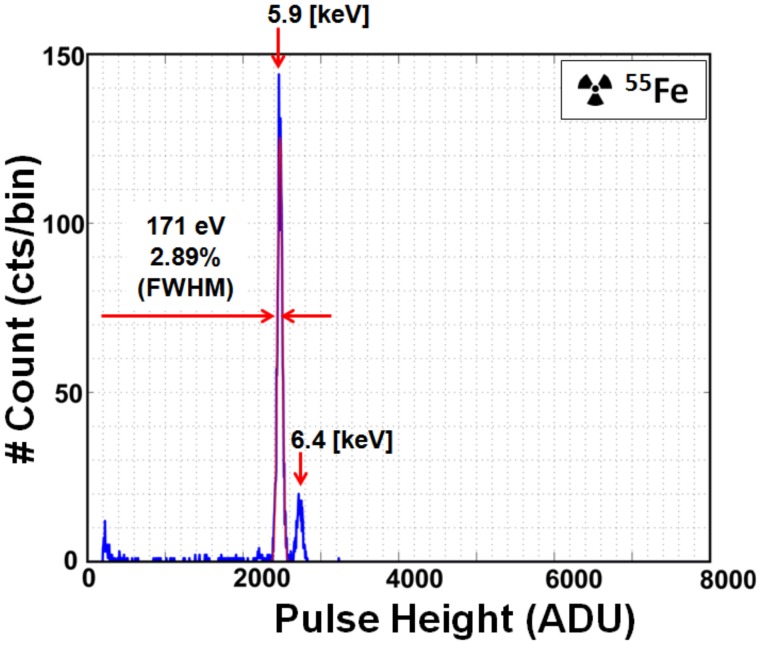
^55^Fe spectrum with a single X-ray event at 16 × 6 pixels at high-gain setting. One bin = 5 ADU. Spectrum is fitted with a Gaussian distribution at the Mn-K_α_ (5.9 keV) line.

The spectral performance is further improved by applying the high-gain setting to the incoming signal. The ^55^Fe spectrum using the high-gain setting is shown in Figure 20. The system-noise reduction and gain correction are used. An Mn-K_α_ peak is obtained at 2516 ADU, corresponding to G_C_ of 186.8 μV/e^−^. Energy resolution is 2.89% (171 eV FWHM) at 5.9 keV.

**Figure 21:**
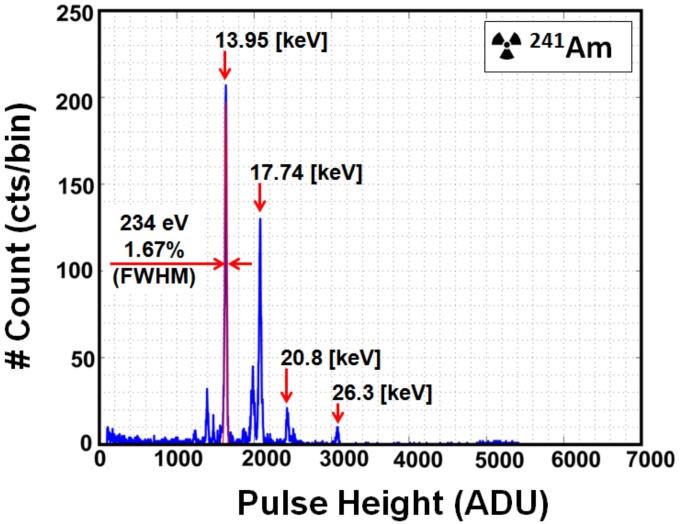
^241^Am spectrum with a single X-ray event at 16 × 6 pixels using low-gain after noise reduction and gain correction. One bin = 3 ADU. Spectrum is fitted with a Gaussian distribution at 13.95 keV. The energy resolution is 1.67% (234 eV FWHM) at 13.95 keV.

X-ray spectra from ^241^Am measured using the low-gain setting are shown in Figure 21. The ^241^Am spectrum has four peaks (13.95 keV, 17.74 keV, 20.8 keV, and 26.3 keV) with the highest count rate at 13.95 keV (1635 ADU). A Gaussian fit near 13.95 keV is done for the estimation of energy resolution. The FWHM is 234 eV at centroid energy (E = 13.95 keV). Thus, the energy resolution corresponds to 1.67% at 13.95 keV.

**Figure 22:**
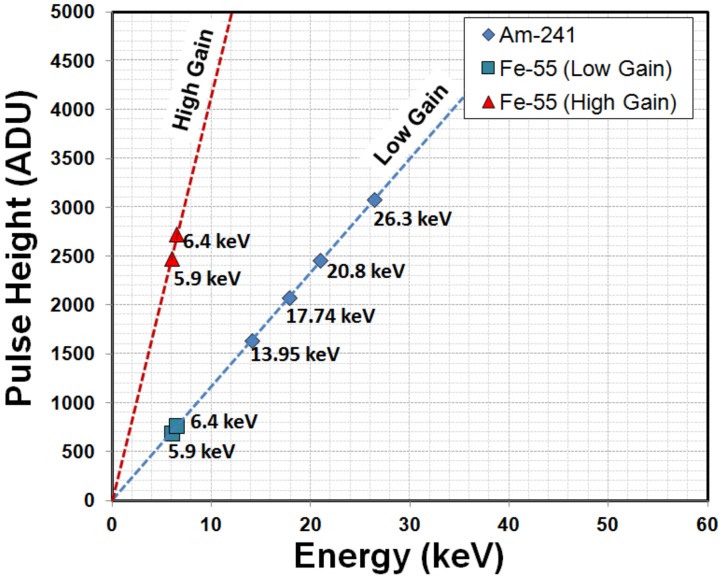
Calibration of the X-ray energy with the signal output. Energy peaks obtained from ^241^Am low-gain setting and ^55^Fe for low- and high-gain settings are fitted with the extrapolated linear line.

Figure 22 shows the pixel output for the equivalent incoming energy using low- and high-gain settings. Extrapolated lines for low-gain (52 μV/e^−^) and high-gain (187 μV/e^−^) determined by the shot-noise measurement of Figure 15 agree well with the X-ray energy spectral peaks obtained from the output voltage peaks of ^55^Fe and ^241^Am sources.

### 5.4. Noise Analysis

**Figure 23:**
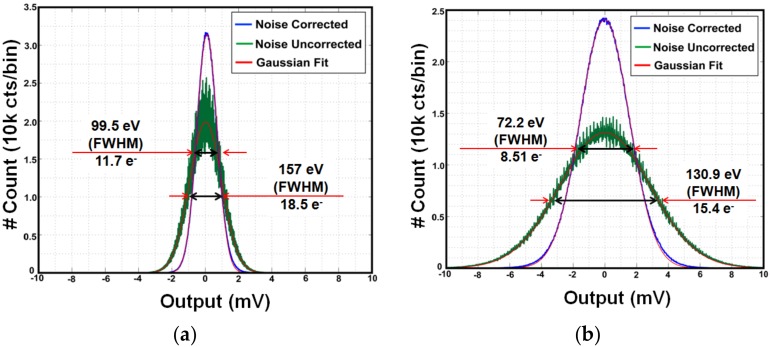
Noise at the pedestal peak of ^55^Fe spectra. (**a**) Noise for low-gain setting before and after system-noise reduction. (**b**) Noise for high-gain setting before and after system-noise reduction. The RMS noise after system-noise reduction is 11.7 e^−^ rms for low-gain and 8.51 e^−^ rms for high-gain.

In order to estimate the readout noise, histograms at the pedestal are plotted as shown in Figure 23a,b for low- and high-gain settings, respectively. Histograms of the input-referred readout noise with and without the system-noise reduction technique at the pedestal peak with ^55^Fe are plotted. Gussian-fitted curves to the system-noise reduced data are also shown. Readout noise of 18.5 e^−^ rms is reduced to 11.7 e^−^ rms using the low-gain setting and the system-noise reduction technique. Noise is further reduced to 8.51 e^−^ rms using the high-gain setting at in-pixel CSA circuit gain configuration.

**Figure 24:**
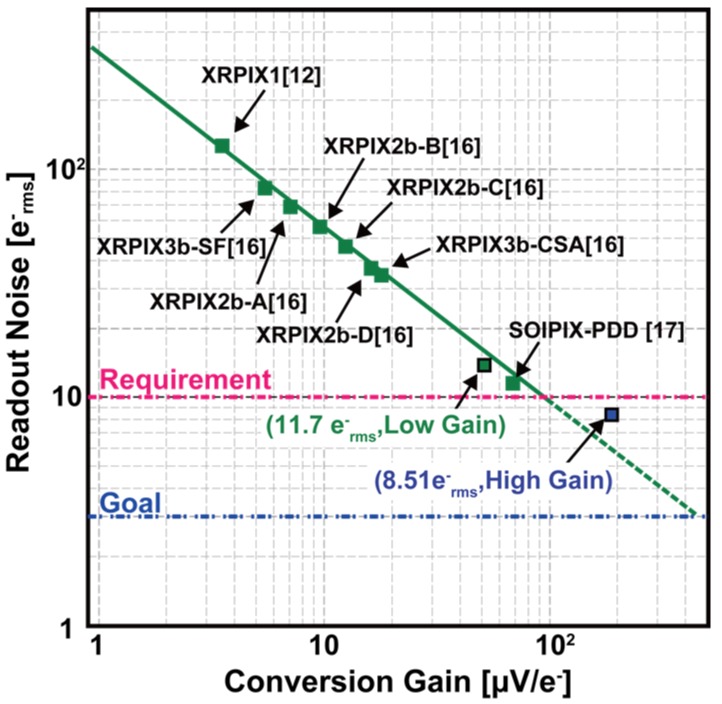
Comparison of noise in the pedestal spectra of ^55^Fe with the noise from our prior work. Input-referred RMS noises are 8.51 e^−^ rms and 11.7 e^−^ rms at low- and high-gain, respectively.

Noise comparison of the designed chip with our prior works of SOI-based X-ray detectors [12,16,17] is shown in Figure 24. Readout noise decreases linearly as the conversion gain increases. Using the high-gain setting, we achieved a noise level better than our requirement. However, reduction in noise is limited to a level no better than the noise level extrapolated by our prior implantations. This may be due to residual system-noise which was minimized but not completely removed by the system-noise reduction technique. With our experiences from prior developments and further improvements in design, the SOI-based X-ray detector technology has a potential goal to reach an ultimate noise level below 3 e^−^ rms. Also, the theoretical fano-limit energy resolution with read noise of 8.51 e^−^ rms at 5.9 keV is 131.2 eV, as opposed to 171 eV obtained in our experiment. This shows that there are unknown factors for improvements in design for obtaining higher energy resolution.

## 6. Conclusions

Table 1 summarizes the performance of the designed chip. The proposed SOIPIX-PDD uses a multi-well structure under the SOI for fast and high charge collection efficiency. The designed prototype chip achieved a very low dark current of 44.8 pA/cm^2^ at −30 °C. With a very low detector node capacitance and in-pixel gain selection configuration, a very high charge-to-voltage gain of 52 µV/e^−^ and 187 µV/e^−^ has been achieved. High-gain and low-gain settings in conjunction were used for the detection of a wide dynamic range of X-ray signals. The improved noise performance is demonstrated using the dual gain CSA pixel. A noise level (8.5 e^−^ rms) below the required target level for X-ray astronomy has been achieved. The energy resolution of 2.89% (171 eV FWHM) at the Mn-K_α_ (5.9 keV) line of ^55^Fe with the high-gain setting has been realized. Also, we are able to successfully detect X-ray energy using ^241^Am radioisotope with very good energy resolution of 1.67% (234 eV FWHM) at 13.95 keV.

With our continuous effort in the design of XRPIX series to improve the energy resolution, readout noise, and other electrical and spectroscopic performance aspects, we believe that the SOI-based monolithic event-driven pixel detector is a viable candidate for next-generation X-ray detectors in future astronomical missions. In our next design, we will modify SOIPIX-PDD to have small node capacitance to reduce the noise. Also, the implementation of on-chip ADC for high speed readout and low noise is possible for high event count rate. Evaluation of radiation hardness and its improvement in future designs are some of the design considerations for our next generation SOIPIX-PDD X-ray detector.

## Figures and Tables

**Table 1:** Performance Summary of Dual Gain CSA SOIPIX-PDD.

Parameter	Value
Process technology	0.2 μm FD-SOI CMOS
Supply voltage	3.3 V, 1.8 V (Analog), 1.8 V (Digital)
BPW pinned voltage	−3.2 V
Back-gate voltage	−60 V
Chip size	2.9 mm × 2.9 mm
Pixel array	16 × 16 (16 × 6 effective array)
Pixel size	40 μm × 40 μm
Substrate thickness	200 μm
Substrate resistivity	>25 kΩcm
Wafer type	Floating zone, p-type (fz-p)
Conversion gain	52 μV/e^−^, 187 μV/e^−^
Dark current density	44.8 pA/cm^2^ at −30 °C
Noise ^a^	8.5 e^−^ rms (High-gain), 11.7 e^−^ rms (Low-gain)
Energy resolution ^b^	^241^Am: 1.67% (234 eV FWHM) at 13.95 keV
	^55^Fe: 2.89% (171 eV FWHM) at 5.9 keV

^a^ Noise after system-noise reduction and gain correction at pedestal peak of ^55^Fe, ^b^ Energy resolution with low-gain setting for ^241^Am, high-gain setting for ^55^Fe.

## References

[1] Gaskin A.J., Harrison A.F. The X-ray surveyor mission: A concept study. Proceedings of the SPIE 9601, UV, X-ray, and Gamma-Ray Space Instrumentation for Astronomy XIX.

[2] Lumb D.H., Berthiaume G.D., Burrows D.N., Garmire G.P., Nousek J.A. (1991). Charge coupled devices (CCDs) in X-ray astronomy. Exp. Astron..

[3] National Aeronautics and Space Administration (2013). Goddard Space Flight Center: X-ray Detectors Electrical Current Detections. https://imagine.gsfc.nasa.gov/science/toolbox/xray_detectors_electric.html.

[4] Murray S., Bautz M., Burrows D., Falcone A., Kenter A., Kraft R. (2011). Active Pixel X-ray Sensor Technology Development for SMART-X Focal Plane. Active Pixel X-ray Sensors. https://beyondeinstein.nasa.gov/studies/rfi/Murray_Active_Pixel_Sensors.pdf.

[5] Hull S.V., Falcone A.D., Burrows D.N., Wages M., Chattopadhyay T., McQuaide M., Bray E., Kern M. Recent X-ray hybrid CMOS detector developments and measurements. Proceedings of the High Energy Astrophysical Phenomena (astro-ph.HE).

[6] Prigozhin G., Suntharalingam V., Busacker D., Foster R.F., Kissel S., LaMarr B., Soares A.M., Villasenor J., Bautz M. (2009). Characterization of three-dimensional-integrated active pixel sensor for X-ray detection. IEEE Trans. Electron Devices.

[7] Lutz G. (2005). DEPFET development at the MPI semiconductor laboratory. Nuclear Instrum. Methods Phys. Res. A.

[8] Kenter A., Kraft R., Gauron T., Murray S.S. Monolithic CMOS Imaging X-ray Spectrometers. Proceedings of the High Energy, Optical and Infrared Detectors for Astronomy VI.

[9] Arai Y., Miyoshi T., Unno Y., Tsuboyama T., Terada S., Ikegami Y., Kohriki T., Tauchi K., Ikemoto Y., Ichimiy R. (2010). Developments of SOI monolithic pixel detectors. Nuclear Instrum. Methods Phys. Res. A.

[10] Arai Y., Miyoshi T., Unno Y., Tsuboyama T., Terada S., Ikegami Y., Kohriki T., Tauchi K., Ikemoto Y., Ichimiy R. (2011). Developments of SOI pixel process technology. Nuclear Instrum. Methods Phys. Res. A.

[11] Nakashima S., GandoRyu S., Tsuru T.G., Taked A., Arai Y., Miyoshi T., Ichimiy R., Ikemoto Y., Imamura T., Ohmoto T. (2012). Progress in development of monolithic active pixel detector for X-ray astronomy with SOI CMOS technology. Technol. Instrum. Part Phys..

[12] Ryu S.G., Tsuru T.G., Nakashima S., Iwata A. (2011). First performance evaluation of an X-ray SOI pixel sensor for imaging spectroscopy and intra-pixel trigger. IEEE Trans. Nuclear Sci..

[13] Ryu S.G., Tsuru T., Prigozhin G.Y., Kissel S., Bautz M.W., LaMarr B., Nakashima S., Foster R.F., Takeda A., Arai Y. (2013). Tests with Soft X-rays of an Improved Monolithic SOI Active Pixel Sensor. IEEE Trans. Nuclear Sci..

[14] Matsumura H., Tsuru T.G., Tanaka T., Takeda A., Arai Y., Mori K., Nishioka Y., Takenaka R., Kohmura T., Nakashima S. (2015). Improving charge-collection efficiency of SOI pixel sensors for X-ray astronomy. Nuclear Instrum. Methods Phys. Res. A.

[15] Tamasawa K., Kohmura T., Konno T., Tsuru T.G., Tanaka T., Takeda A., Matsumura H., Mori K., Nishioka Y., Takenaka R. Study of the basic performance of the XRPIX for the future astronomical X-ray satellite. Proceedings of the International Workshop on SOI Pixel Detectors (SOIPIX2015).

[16] Takeda A., Tsuru T.G., Tanaka T., Uchida H., Matsumura H., Arai Y., Mori K., Nishioka Y., Takenaka R., Kohmura T. (2015). Improvement of spectroscopic performance using a charge-sensitive amplifier circuit for an X-ray astronomical SOI pixel detector. J. Instrum..

[17] Kamehama H., Kawahito S., Shrestha S., Nakanishi S., Yasutomi K., Takeda A., Tsuru T.G., Arai Y. (2018). A low-noise X-ray astronomical silicon-on-insulator pixel detector using a pinned depleted diode structure. Sensors.

[18] Kamehama H., Kawahito S., Shrestha S., Arai Y. Fully depleted SOI pixel photo detectors with backgate surface potential pinning. Proceedings of the International Image Sensor Workshop.

[19] Tsuru T.G., Matsumura H., Takeda A., Tanaka T., Nakashima S., Arai Y., Mori K., Takenaka R., Nishioka Y., Kohmura T. Development and performance of kyoto’s X-ray astronomical SOI pixel (SOIPIX) sensor. Proceedings of the SPIE, Instrumentation and Methods for Astrophysics.

[20] Shrestha S., Kamehama H., Yasutomi K., Kawahito S. Event-Driven Dual-Gain Fully-Depleted SOI Based X-ray Detector for High Energy Particle Imaging. Proceedings of the 2017 International Image Sensor Workshop (IISW).

